# Diet Quality and Food Prices Modify Associations between Genetic Susceptibility to Obesity and Adiposity Outcomes

**DOI:** 10.3390/nu12113349

**Published:** 2020-10-30

**Authors:** Hannah Yang Han, Catherine Paquet, Laurette Dubé, Daiva E Nielsen

**Affiliations:** 1 School of Human Nutrition, McGill University, Sainte-Anne-de-Bellevue, QC H9X 3V9, Canada; yang.han3@mail.mcgill.ca; 2 Faculté des Sciences de l’Administration, Université Laval, Québec, QC G1V 0A6, Canada; catherine.paquet@fsa.ulaval.ca; 3 Australian Centre for Precision Health, University of South Australia, Adelaide 5001, Australia; 4 Desautels Faculty of Management, McGill University, Montreal, QC H3A 1G5, Canada; laurette.dube@mcgill.ca

**Keywords:** gene-environment interaction, food environment, in-store retail food environment, diet quality, polygenic risk, obesity

## Abstract

The role of the retail food environment in obesity risk is unclear, which may be due in part to the lack of consideration of individual differences in the responsivity to food cues. This cross-sectional investigation geo-temporally linked the CARTaGENE biobank (including genetic, dietary, lifestyle, and anthropometric data) with in-store retail food environment data to examine interactions between a polygenic risk score (PRS) for obesity and (1) diet quality (*n* = 6807) and (2) in-store retail food measures (*n* = 3718). The outcomes included adiposity-related measures and diet quality assessed using the 2010 Canadian-adapted Healthy Eating Index. A vegetable:soft drink ratio was constructed for each retail measure to assess the relative healthfulness of exposures. Generalized linear models adjusted for individual and neighborhood socio-demographic factors were used to evaluate main and interactive effects. Diet quality significantly modified the association between polygenic risk of obesity and body mass index, waist circumference, and body fat percent. A significant interaction was also observed between PRS and regular price of vegetables in relation to soft drinks on waist circumference. These results replicate previous reports of diet moderating polygenic risk of obesity and suggest that prices of low vs. high-energy density foods are an intervention target to address population obesity rates.

## 1. Introduction

Obesity is a complex phenotype influenced by genetic, behavioral, and environmental factors [1]. Excess adiposity increases the risk of nearly every chronic condition such as diabetes, heart disease, cancer, and poor mental health and may ultimately decrease one’s lifespan [1]. 

Ingestive behavior is a significant contributor to obesity, with consumption of energy-dense, highly palatable foods being recognized as “problem foods” for weight loss [2]. The modern-day obesogenic food environment poses challenges for healthful eating, with growing evidence implicating the ubiquitous presence of highly palatable foods and food cue stimuli as a contributor to eating behavior and weight gain [3]. As the primary locations for purchasing food, retail food environments may influence the public’s ingestive behaviors and may contribute to obesity risk through the food options available and corresponding financial/marketing factors [4]. Indeed, consumer choices and purchasing intentions are known to be influenced by marketing strategies including price, promotion, shelf display, brand availability, and nutrition labeling [5].

The retail food environment assessed by residential proximity to food retailers and exposure to food marketing may associate with anthropometric outcomes (body mass index (BMI) and waist circumference) by affecting food availability and food choices [6]. However, several null findings have been reported among investigations of retail food environments and obesity-related outcomes [6,7]. The conflicting evidence may be due to a lack of precision in defining the in-store retail food environment and a lack of consideration of inter-individual differences in the responsivity to food cue stimuli that are encountered within retailers. Methods that take into account both within store food options and in-store marketing strategies may provide more robust measures of retail food environments, particularly as the role of food marketing in influencing food choices is increasingly acknowledged [8]. The “Four P’s of marketing” (product, price, promotion, and place), commonly evaluated in marketing research, represents an actionable target for investigating associations between the retail food environment and diet/health outcomes that may improve the measurement precision of in-store retail food environments. 

Separate from environmental factors, BMI is known to be heritable and genome-wide association analyses have identified a number of common BMI susceptibility loci [9]. When aggregated, these variants account for only 2.7% of variation in BMI [9], indicating a potential role for interactions between genetic variants and environmental exposures on obesity-related outcomes. Indeed, previous studies have reported that adherence to healthy dietary patterns and limiting consumption of sugar sweetened beverages and fried food might attenuate the genetic risk associated with elevated BMI [10,11,12]. One recent study reported a significant interaction between genetic risk to obesity and proximity to fast-food outlets on BMI [13]; however, no study has evaluated the interactions between everyday exposures to food cues in the in-store retail food environment and genetic susceptibility to obesity on diet and anthropometric outcomes. Some of the variants implicated in genetic risk for obesity are involved in neurobehavioral circuits related to dopamine reward and food cue responsivity, suggesting that exposures to in-store retailer food cue stimuli may affect ingestive behavior in distinct ways according to genetic background. Therefore, the primary objective of this investigation was to assess the main and interactive associations between exposures in the retail food environment and a polygenic risk score (PRS) for obesity on diet and adiposity-related outcomes using data from a Canadian biobank linked with geographical data on in-store retail food environment exposures. We also replicate the interaction between diet quality and adiposity-related outcomes according to genetic susceptibility, which has been reported previously [10].

## 2. Materials and Methods 

### 2.1. Study Design and Population

Cross-sectional anthropometric, dietary (collected during 2012), lifestyle, and genetic data collected between 2008–2013 were obtained from the CARTaGENE biobank, a Quebec (Canada) population prospective cohort designed to investigate environmental, lifestyle, and genomic determinants of chronic diseases [14]. The genotyped subset of the biobank is currently comprised of 12,111 adults aged 40–69 years old from six regions of the province: Sherbrooke, Saguenay, Quebec City, the Greater Montreal Area, Gatineau, and Trois-Rivieres. The present analysis for diet quality-PRS interactions on adiposity-related outcomes included *n* = 6087 participants who had diet, genetic, and anthropometric data available (“diet sample”). Upon linkage with data on the Quebec retail in-store food environment, *n* = 3718 remained (“retail sample”, see Data Linkage and Figure S1). The study was approved by CARTaGENE’s Sample and Data Access Committee and ethics approval was obtained from the McGill University Faculty of Agriculture and Environmental Sciences Research Ethics Board. 

### 2.2. Genotyping and Genetic Risk Score

Participants were genotyped with the Infinium Omni 2.5M Array, the UK Biobank Axiom Array or the Illumina Infinium Global Screening Array and genetic imputation was performed using the Michigan Imputation Server. Detailed quality control and imputation steps are available at https://www.cartagene.qc.ca/en/researchers/catalogue/genetic-data. Principal components analysis was conducted on genotyping data in PLINK 1.9 with the *-pca* function to account for population stratification in analyses [15]. The eigenvalues indicated that the first three principal components captured sufficient variance in the data while reducing the dataset’s dimensionality, and were thus used as covariates for ancestry in statistical analyses.

We derived a previously described PRS for obesity from 97 BMI-associated single nucleotide polymorphisms (SNPs) identified in a genome-wide association study (GWAS) (Table S1) [9]. The resulting PRS ranged from 0–194 with each unit corresponding to one risk allele. A higher score indicates a higher genetic predisposition to risk of obesity. Where sample size permitted, the PRS was examined by quintiles to compare associations between the extremes of polygenic risk categories (quintile one vs. quintile five).

### 2.3. Assessment of Healthy Dietary Pattern 

Usual dietary intake over the previous 12 months was assessed in 2012 using the Canadian adaptation of the Diet History questionnaire, a semi-quantitative food frequency questionnaire comprised of 164 food and beverage items. The Diet History questionnaire has been validated for cross-sectional assessment of dietary intake [16].

Diet quality was calculated with the Canadian adaptation of the Healthy Eating Index 2010 (HEI-C). The HEI-C consists of 11 dietary components: eight adequacy components and three moderation components [17]. Those components sum-up to a continuous score ranging from 0–100, with a higher score reflecting healthier diet quality and associated with lower likelihood of obesity [17]. The standard scoring is based on age- and sex- specific serving recommended by the 2007 Canada’s Food Guide. 

### 2.4. Assessment of Retail Food Environment

Retail data was obtained from a marketing database (Nielsen Corporation) that contains weekly purchase and marketing information on consumer-packaged goods and fresh produce sold within a representative sample of Quebec retailers during the years 2008–2013. This retail data has been used by others to develop a method for community-level nutrition monitoring in Montreal, Quebec [18]. Grocery stores, mass merchandisers, and convenience stores are represented in the data and are classified based on *n* = 227 forward sortation areas (FSA) in the province of Quebec (Figure S2). An FSA is a geographical unit indicated by the first 3 digits of a postal code. It represents part of a major metropolitan area, a medium-sized city, or a specific rural region [19]. The marketing database included every Universal Product Code (UPC), an identifier of a unique product, within different food categories and each UPC’s packaging (item description, brand name, package size, the number of individual items within the pack) and marketing information (weekly price and in-store promotion). 

Methods outlined by Ma et al. were used to derive retail food environment measures that reflect the “Four Ps of marketing” [20]: Product as a quality indicator that classifies food groups into healthy (vegetable) or unhealthy (soft drinks); Price as an affordability indicator that includes regular and discounted price; Promotion as a marketing effort indicator that captures retailers’ attempts to improve food product awareness through non-price promotion (e.g., in-store product displays); Place as an availability indicator that reflects the variety of available food at point of purchase. The detailed information on retail food environment measures used in this investigation (product variety, regular price per serving, discount frequency, and in-store food displays) has been described previously [21]. A ratio of marketing indicators for vegetables and soft drinks was calculated as a measure of healthful to unhealthful retail exposure. A ratio above 1 was considered to represent a healthful in-store environment for the product variety, discount frequency, and in-store food display indicators (i.e., vegetables were of a greater variety, more often on sale, and more often visibly on display compared to soft drinks). Conversely, a ratio below 1 represented an unhealthful in-store environment for these retail indicators. For regular price, a ratio above 1 indicated that the price of vegetables is higher relative to the price of soft drinks, which was considered as an unhealthful in-store environment. While the marketing database consists of a variety of food categories, vegetables and soft drinks were selected for analysis, because they are common targets for dietary intervention, have established beneficial (vegetables) and adverse (soft drinks) links with BMI, and had sufficient data across the marketing variables of interest to the present investigation. 

### 2.5. Linkage of Retail Food Environment Data

CARTaGENE biobank and the marketing database were linked based on year and quarter of data collection (as a proxy for season) and FSA to estimate neighborhood in-store retail food environment exposures. Since UPC availability for the food categories varied throughout 2008–2012 (up to the year of dietary assessment in CARTaGENE), and CARTaGENE data was obtained from 2008–2013 (excluding dietary assessment), exponential smoothing was performed to weight more recent data more heavily than the older data when averaging over the full period of time. This enabled complete usage of marketing data. Approximately 80% of FSAs from the retail dataset were represented in CARTaGENE, providing 5147 participants after data linkage. The combined data was further linked to Canadian Census data (years 2006 and 2011) by census tracts to be able to account for neighborhood demographic factors that have been associated with differences in neighborhood retail food environments: population density (2011 Census), neighborhood prevalence of low-income households, median household income, percent immigrant status, employment rate, and proportion of high school completion (all remaining derived from 2006 Census as these were not assessed in 2011) [6]. Since 1429 participants did not provide a postal code (prohibiting linkage by census tract), the final sample size was 3718.

### 2.6. Assessment of Body Mass Index, Waist Circumference, Percent Body Fat, and Covariates

Information on socio-demographics (age, sex, income, education, and language of study completion) and lifestyle risk factors were obtained from participant questionnaires. Participant weight, height, waist circumference, and percentage (%) of body fat were directly assessed during non-invasive physical examination at biobank assessment centers. Further details on assessment methods have been published previously [14]. BMI was used to categorize participants into underweight (<18.5), healthy weight (18.5–24.9), overweight (25.0–29.9), or obese (≥30.0). Participants with waist circumference >102 cm (for males) or >88 cm (for females) were considered at increased obesity-related health risk [22]. Based on cut-offs suggested by Romero-Corral and colleagues, participants with % body fat >25% (for males) or >35% (for females) were considered to be obese [23].

Previous research recommends adjustment for energy misreporter status (rather than excluding misreporters) when obesity-related variables are the outcomes of interest, since overweight/obese individuals tend to under-report their energy intake [24]. Participants were categorized into under-, plausible-, and over-reporter following the method described previously [21,24].

### 2.7. Statistical Analysis

Multiple imputation by chained equation was conducted to minimize the risk of bias owing to missing data for the environmental exposures, covariates, and outcomes using MI and MIANALYZE Procedure in SAS 9.4 (SAS Institute Inc., Cary, NC, USA). Data were missing at random and so imputation was performed according to standard approaches, which entailed 20 times for PRS × HEI-C interaction analysis (9% of participants missing necessary values) and 50 times for PRS × retail food environment analysis (49% of participants missing necessary values), due to higher percentage of missing values in retail food environment measures [25]. All variables from the statistical model were included for the imputation. Individual measures for vegetables and soft drinks (i.e., variety, display, price, and discount frequency) were included as auxiliary variables. Exposure measures were standardized by subtracting the mean and dividing by the standard deviation to facilitate interpretation.

Generalized linear models were conducted to assess the main effect associations of standardized PRS and HEI-C (and its individual components) with waist circumference, BMI, and % body fat, and the associations of standardized HEI-C with the adiposity-related outcomes according to PRS quintiles. Standardized HEI-C and PRS (as standardized continuous score and quintiles) interactions were assessed by including an interaction term in the model (PRS × HEI-C) while keeping the main effects. Because previous reports have suggested that genetic susceptibility to obesity may differ by sex, or exhibit a peak effect at different stages of the life course between males and females, analyses were stratified by sex [26]. Models were adjusted for age, sex (when not used to stratify analyses), the first three principal components of ancestry, marital status, household income, education, smoking status, total energy intake, alcohol consumption, physical activity level, misreporting status, and language (English or French) and season in which the questionnaire was completed, and source of genotyping. Covariates were related to either self-reported dietary intake or the adiposity outcomes of interest. Since energy intake may be along the causal pathway between the PRS for obesity and adiposity-related outcomes, we performed sensitivity analyses that excluded total energy intake from the model. 

The analysis was repeated for PRS and retail food environment interactions accounting for spatial clustering by FSA through the use of generalized estimating equations. HEI-C and adiposity-related outcomes were the outcomes of interest for this analysis. In addition to the covariates previously described, neighborhood sociodemographic factors from Census data were also included as covariates (outlined in Linkage of Retail Food Environment Data Section). Analysis of PRS in quintiles was not conducted due to the reduced available sample size. All reported *p*-values are two-sided with alpha level of 0.05. Analyses were conducted in SAS 9.4 using GENMOD Procedure (SAS Institute Inc., Cary, NC, USA).

## 3. Results

### 3.1. Participant Characteristics

Table 1 depicts participant characteristics of the analytical sample. Participants’ mean age was approximately 55 years and 54% were female. The majority completed the study questionnaires in French. The proportion of plausible and under-reporters of energy was roughly equal. Approximately 45% of the analytical sample reported earning an annual household income of >$75,000 CAD and had a university degree (Bachelor’s or higher). HEI-C score ranged from 10–92 and the PRS ranged from 64–110. The mean BMI was 27.3 kg/m^2^ and 25% of participants were considered obese based on BMI (≥30 kg/m^2^). The mean waist circumferences for males and females were 99 cm and 88 cm, respectively. The mean % body fat for males and females were 25% and 35%, respectively. Although males and females had similar PRS means, based on BMI, the proportion of obese participants was higher among males compared to females (males: 26% vs. females: 23%, *p* = 0.0038). However, based on waist circumference and % of body fat, the proportion of participants at increased obesity-related health risk was higher among females (waist circumference: males 36% vs. females 42%, *p* < 0.0001; % body fat: males 45% vs. females 49%, *p* = 0.0022). 

Table 2 depicts the least squares means adjusted for age, sex, total energy intake, and energy reporter status of HEI-C individual components per HEI-C quartile. A higher diet quality score was associated with higher consumption of adequate components and lower consumption of moderation components with the exception of refined grains and sodium intake.

Compared to the lowest PRS quintile, participants with increasing PRS quintiles had higher waist circumference, BMI, and % body fat (Table 3). PRS quintiles were not associated with overall HEI-C score, but were significantly associated with the individual component for fruit and vegetable intake such that intake tended to decrease as PRS increased (Table 4).

### 3.2. Dietary Association with Risk of Obesity According to Genetic Risk Score

Significant main effect associations were observed between PRS and adiposity-related outcomes: 1 SD increase in PRS was associated with 1.3 cm, 0.6 kg/m^2^, and 0.6% increase in waist circumference, BMI, and % body fat, respectively. 1 SD increase in HEI-C was associated with a 1.6 cm, 0.5 kg/m^2^, and 0.7% decrease in waist circumference, BMI, and % body fat, respectively.

A significant PRS × HEI-C interaction was observed on adiposity-related outcomes (*p*_waist circumference_ = 0.005; *p*_BMI_ = 0.039; *p*_% body fat_ = 0.041): the negative interaction estimates suggest that the inverse associations between HEI-C and adiposity-related outcomes are strengthened for participants with higher PRS (Table 5). The analysis with PRS quintiles generated similar patterns for the main effect associations of PRS and HEI-C with BMI, waist circumference, and % body fat. The interaction terms were non-significant when PRS was categorized as quintiles. 

**Figure 1:**
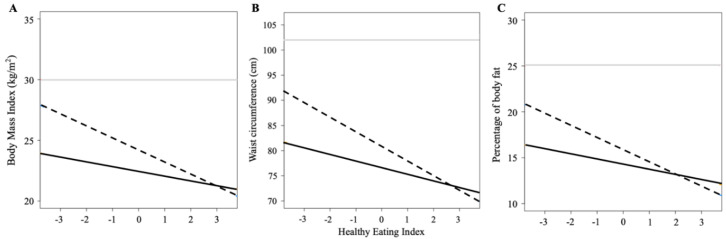
Estimated waist circumference and BMI for dietary quality score in male participants. Interaction plots showing the association between standardized Healthy Eating Index 2010 and (**A**) body mass index, (**B**) waist circumference, and (**C**) percentage of body fat among males in the first quintile (solid black line) and last quintile (dashed black line) of polygenic risk score of obesity. The solid grey line represents the cut-off value for obesity (**A**) or increased obesity-related health risk (**B**) according to body mass index or waist circumference or percentage of body fat.

Table 5 also displays results of the analysis stratified by sex. The main effect associations of HEI-C and PRS with adiposity-related outcomes were statistically significant for both males and females. PRS×HEI-C interactions were only significant among males (*p*_waist circumference_ = 0.0004; *p*_BMI_ = 0.0013; *p*_% body fat_ = 0.0049), with the estimates almost doubled compared with the results of the non-stratified analysis. When quintiles of PRS were assessed in males, an increase in every 1 SD of HEI-C was negatively associated with all adiposity-related outcomes, and such associations were more pronounced among participants with highest genetic risk (PRS quintile 5) compared with those with the lowest genetic risk (PRS quintile 1) (Figure 1).

The sensitivity analysis excluding total energy intake shows similar results for PRS and interaction associations. The main effects of HEI-C on adiposity-related outcomes were all attenuated (Table S2). 

### 3.3. Associations of In-Store Retail Food Measures with Risk of Obesity According to PRS

The participant characteristics of the analytical sample with retail food environment data did not differ from the characteristics of the larger sample (Tables S3–S5). Table 6 reports the main effect associations between PRS and retail measures with waist circumference, BMI, and % body fat. While the PRS was significantly associated with the adiposity-related outcomes (but not diet quality), the in-store retail measures were not associated with either the adiposity-related outcomes or diet quality. A significant interaction between PRS and regular price was observed on waist circumference (*p*_regular price_ = 0.031). The positive interaction suggests that the association between regular price and waist circumference is accentuated as the genetic susceptibility to obesity increases. 

When analyses were stratified by sex, a significant main effect association was observed between the display ratio and both waist circumference and BMI among males (Table 7). A significant PRS × discount ratio interaction on the adiposity-related outcomes was also observed among males. The negative interaction suggests that higher discount frequency of vegetables compared to soft drinks associates with lower waist circumference, BMI, and % body fat as genetic susceptibility to obesity increases. Among females, a significant main effect association was observed between regular price and HEI-C (Table 8). A positive PRS × regular price ratio on % body fat was observed such that the higher regular price of vegetables compared to soft drinks may associate with higher % body fat. 

## 4. Discussion

Both diet quality and polygenic risk of obesity were independently associated with BMI, waist circumference, and % body fat, and the interactive effect suggests that individuals at high polygenic risk may be most responsive to a healthy diet. Epidemiological evidence suggests that improved overall diet quality is associated with reduced long-term body weight and weight gain, likelihood of obesity, and risk of death [17,27,28]. Recent analyses have shown that poor adherence to a healthy dietary pattern and a higher consumption of unhealthy dietary factors including sugar-sweetened beverages and fried foods accentuated genetic susceptibility to obesity assessed using BMI [10,11,12]. However, a meta-analysis with 18 European ancestry cohorts suggested nominal evidence of interaction between a PRS based on 14 variants and diet quality on waist-to-hip ratio [29]. The limited number of variants in that study might partially explain the lower magnitude of observed effect size.

Main effect associations with price or discount frequency were observed among both sexes in the present investigation. The sex-specific main effect associations for in-store display suggest that males and females respond differently to visual in-store retail food cues. In-store nutrition interventions, such as price discounts and vouchers, are generally effective in promoting purchasing of healthy foods, which may also relate to consumption [8]. Such price interventions could be effective particularly for promoting nutrition among children and low socio-economic status populations [30]. Product placement and visual displays of food in stores may also influence purchasing by attracting consumer’s attention [5]. While some studies have reported associations between in-store food environment measures and dietary and obesity-related outcomes, several null findings limit the overall evidence level [7]. Our results suggest that the mixed evidence could be due to the lack of considering inter-individual variability in responsivity to food cues in the retail environment.

The average regular price of vegetables relative to soft drinks was observed to modify the association between PRS and waist circumference. This finding is notable, since price of food is a widely recognized barrier for adherence to a healthy diet [31]. The positive interaction indicates that those with higher genetic risk of obesity might be more vulnerable to the barriers associated with higher prices of low (e.g., vegetables) relative to high (e.g., soft drinks) energy-dense foods, which may influence adiposity outcomes by acting as a determinant of food choice. Reduced prices of low-energy dense foods may provide motivation to purchase and subsequently consume these foods, which may be intrinsically less appealing to individuals with higher polygenic risk for obesity. A recent study reported an interaction between genetic risk to obesity and proximity to fast-food outlets on BMI [13]. Although the food environment was evaluated with a different measure, the result adds support to our findings of a moderating effect of the food environment on genetic susceptibility to obesity. 

Our results from the sex-stratified analysis support an interactive association between PRS and price on adiposity-related outcomes among both sexes. These findings are in line with our previous investigation that reported an interaction between price and a genetic variant near a dopamine gene receptor on diet quality [21]. Financial decisions are encountered on a daily basis, with budgets for food spending representing a common consideration in the population. One’s willingness and ability to pay for food varies according to socioeconomic status and personal food preferences/values. Indeed, an investigation conducted with the UK Biobank reported that socio-economic position may best capture the environmental component that accentuates the risk of obesity in adults with genetic susceptibility [32]. 

We observed that the cumulative effect of the 97 BMI-loci in the PRS was associated with fruit and vegetable intake, although not with overall diet quality or other individual components. Individual SNPs in the PRS such as *BDNF*, *MC4R*, *GRID1*, *PARK2* are involved in appetite and food behavior phenotypes [33]. Repeated exposures to and consumption of highly palatable foods have been linked with cravings and addiction-like eating behaviors [34]. Food and food-related cues, such as food marketing, can stimulate dopamine release and activate reward-related brain circuits that are prominent in the mesolimbic dopamine pathway [34]. Moreover, a recent study that evaluated 106 BMI-related SNPs identified the top 25 susceptibility genes with highest expression level and specificity in brain regions involved in addiction and reward [34]. Twenty-three of those SNPs were part of the obesity PRS used in this present study (Supplemental Table S6). Thus, it is important to note that GWAS-significant loci implicated in BMI likely relate to adiposity outcomes through influences on both metabolism (physiological breakdown and use of energy in the body) and dietary behaviors (food preferences and food cue reactivity). The impact of genetic variation on ingestive behaviors therefore represents a target for future investigations to better understand the initial action of food intake that impacts downstream effects of metabolism on obesity risk and related morbidity. 

Sex differences were apparent among our observed associations, which may be a result of sex-specific hormonal profiles, differences in reward circuitry and food cue responsivity, metabolism, nutrient requirements, and/or genetic profiles [26,35]. Such differences not only lead to distinct sex-specific anthropometric traits and risk factors for obesity, but may also influence how the sexes respond to similar environmental factors [35]. In the present sex-stratified analysis, the observed PRS×diet quality interactions on waist circumference and BMI was driven by males. It is unclear whether this represents a true biological difference between males and females, or if the higher variability in diet quality and anthropometric measures among males provided greater statistical power to detect a statistically significant interaction.

The overall strengths of this study include the availability of biobank data with existing genetic and dietary intake data, direct physical measurements of adiposity-related outcomes, and spatiotemporal alignment with in-store retail food environment measures. There are also limitations. Diet was assessed using a self-reported method, which is prone to measurement error although the use of a diet quality score focused on assessing a pattern of dietary intake rather than nutrient intake alone [36]. Our findings are only generalizable to our study population which was mainly comprised of middle-aged Caucasians, middle class, and educated participants. We observed overall concordance in subject characteristics (age, sex, BMI, annual income, BMI, and physical activity level) between our two analytical samples, and with the overall CARTaGENE biobank sample, with the exception that our samples were comprised of more Caucasian participants [14], suggesting that some selection bias may have occurred, although unlikely related to the outcomes of interest. We performed our retail food environment analyses on imputed data; however, data was missing at random and a recent simulation study concluded that unbiased results can be obtained even with large proportions of missing data, provided that data are missing at random and the imputation model is properly specified [37]. Moreover, the results of a complete case analysis are similar to results from the imputed data (available upon request). The results of our in-store retail food environment analysis used data from 2008–2012. While more recent data sources for linkage were not available, the relationships between food environments and health-related outcomes are not anticipated to change, and recent work has reported that the density of residential supermarkets remained relatively constant from 1971–2008 in four US towns [38]. Similar to this, due to differences in Census methodologies, regional socioeconomic status variables were mostly obtained from 2006 Census as opposed to 2011 which would have more closely matched the time of dietary assessment in the present study. While our in-store retail environment assessment utilized four marketing indicators, we focused on only two food categories. Future efforts are needed to more comprehensively define the in-store retail food environment. It is also important to note that retail food environment data does not capture food intake, although associations between food purchasing data and diet quality at household-level have been reported [39,40]. Moreover, in the present investigation, data linkage was facilitated with FSA and census tract-level variables, thus, some error may be present in the linkage between the retail food environment data and individual data. Finally, our analyses were conducted in only one cohort and replication of our results is required.

## 5. Conclusions

Our findings reflect the potential of a healthful dietary pattern to offset predisposed genetic risk to obesity and highlight the importance of population adherence to a healthy diet, which may be impacted by interventions that target prices of food. In addition, with growing interest in the clinical application of PRS, disclosing one’s polygenic risk for obesity may be useful to improve interventions that target individual ingestive behavior. Indeed, consistent evidence from randomized controlled trials reports positive dietary behavioral outcomes following disclosure of genetic information related to nutrition [41,42]. Future investigations that assess the relationships between genetic variants implicated in ingestive behaviors are warranted in order to better understand the role of genetics at the initial dietary action that impacts downstream obesity risk and to inform intervention approaches.

## Figures and Tables

**Table 1:** Participant characteristics (*n* = 6087).

Characteristic	Descriptive Statistics ^a^
Age, years	55 (8)
Female, n (%)	3307 (54.33)
Household Income, (n (%))	
<CAD 25,000	475 (7.80)
CAD 25,000–50,000	1270 (20.86)
CAD 50,000–75,000	1334 (21.92)
CAD 75,000–150,000	2027 (33.30)
>CAD 150,000	725 (11.91)
Missing	256 (4.21)
Education, (n (%))	
High school or less	1310 (21.52)
College	1968 (32.33)
University or higher	2799 (45.98)
Missing	10 (0.16)
Language (French), n (%)	5784 (95.02)
Ethnicity (Caucasian), n(%)	5787 (96.05)
Physical activity level	1.48 (0.36)
Smoking status, n (%)	
Never	2523 (41.45)
Daily	695 (11.42)
Occasionally	226 (3.71)
Past	2635 (43.29)
Missing	8 (0.13)
Marital status, n (%)	
Married	4062 (66.73)
Divorced	1180 (19.39)
Single	837 (13.75)
Missing	8 (0.13)
Season, n (%)	
October–March	3419 (56.17)
April–September	2664 (43.77)
Missing	4 (0.07)
Waist Circumference (cm)	92.9 (14.4)
Body mass index (kg/m^2^)	27.4 (5.2)
Percentage of body fat	30.6 (8.7)
Genetic risk score	87.18 (6.33)
Energy intake (kcal/day)	1716(961)
Alcohol consumption (kcal/day)	82 (186)
Energy reporter status, n (%)	
Under reporter	2805 (46.80)
Plausible reporter	2946 (48.40)
Over reporter	336 (5.52)
Diet quality scores (min.–max.)	
HEI C2010 score (0–100)	57.7 (13.1)
Adequacy sub-score (0–60)	32.4 (11.9)
Moderate sub-score (0–40)	25.3 (5.2)

CAD: Canadian dollar; HEI-C: Canadian adaptation of the Healthy Eating Index 2010. ^a^ Values are mean (standard deviation) unless otherwise indicated.

**Table 2:** Mean values of Healthy Eating Index 2010 (HEI-C) individual components and overall score presented by HEI-C quartile among diet sample (*n* = 6087).

Components	Standard for Max. Score	HEI-C 2010 Score Quartile (Poorest to Highest Quality)				*p*-Value for Trend
		1	2	3	4	
HEI-C 2010 Score, Ranges	0–100	≤51.8	51.8–60.6	60.6–66.6	≥66.6	
Fruit and vegetables, servings/day	7–8	5.1	6.8	8.0	8.9	<0.0001
Whole fruit, servings/day	1.47–1.68	1.3	1.9	2.6	3.2	<0.0001
Greens and beans, servings/day	0.735–0.84	0.5	0.9	1.3	1.5	<0.0001
Whole grains, servings/day	3–4	0.4	0.5	0.5	1.0	<0.0001
Dairy, servings/day	2–3	1.7	1.7	1.8	1.8	0.0199
Total protein foods, servings/day	2–3	1.3	1.5	1.8	2.0	<0.0001
Seafood and plant protein, servings/day	0.64–0.96	0.2	0.3	0.5	0.8	<0.0001
Fatty acids, (PUFA + MUFA)/SFA	2.5	1.3	1.8	2.0	2.2	<0.0001
Refined grains, servings/day	<50% refined grains	2.2	2.6	2.7	2.3	<0.0001
Sodium, mg/day	AI	2164	2419	2496	2379	<0.0001
Empty calories, % Energy	≤19% of energy	27.1	16.7	13.5	11.3	<0.0001

Least squares mean values are adjusted for sex, age, total energy intake, and reporter status. Servings correspond to Canada’s Food Guide 2007 serving. HEI-C: Canadian adaptation of the Healthy Eating Index 2010; AI, adequate intake; PUFA: polyunsaturated fatty acids; MUFA: monounsaturated fatty acids; SFA: saturated fatty acids.

**Table 3:** Mean and standard deviation of HEI-C, waist circumference, body mass index (BMI), and percentage of body fat by polygenic risk score (PRS) quintile among diet sample.

PRS Quintile	1	2	3	4	5	*p*-Value for Trend
Combined, *n* = 6087						
HEI-C	58.1	57.0	57.6	57.5	57.3	0.2609
Waist circumference (cm)	90.9	92.8	93.8	94.1	95.0	<0.0001
BMI (kg/m^2^)	26.3	27.1	27.6	27.7	28.3	<0.0001
percentage of body fat	29.0	30.0	30.6	30.6	30.9	<0.0001
Male, *n* = 2780						
HEI-C	55.2	54.7	54.9	55.2	54.1	0.6340
Waist circumference (cm)	96.1	98.3	99.8	99.4	101.0	<0.0001
BMI (kg/m^2^)	26.9	27.6	28.3	28.3	28.8	<0.0001
percentage of body fat	24.1	24.9	25.7	25.3	25.9	<0.0001
Female, *n* = 3307						
HEI-C	60.9	59.4	60.5	59.7	60.3	0.1912
Waist circumference (cm)	85.7	87.1	88.0	88.7	89.2	<0.0001
BMI (kg/m^2^)	25.8	26.6	27.0	27.1	27.8	<0.0001
percentage of body fat	33.9	35.1	35.5	35.7	35.8	<0.0001

PRS, polygenic risk score for obesity; HEI-C, Canadian adaptation of the Healthy Eating Index 2010; BMI, Body Mass Index.

**Table 4:** Mean of HEI-C individual components by PRS quintile among diet sample.

PRS Quintile	1	2	3	4	5	*p*-Value for Trend
Combined, *n* = 6087						
Fruit and vegetables, servings/day	7.2	6.9	7.3	6.8	6.9	0.0409
Whole fruit, servings/day	2.2	2.0	2.3	2.1	2.1	0.0052
Greens and beans, servings/day	1.0	1.0	1.0	0.1	1.0	0.6220
Whole grains, servings/day	0.7	0.6	0.6	0.6	0.6	0.4685
Dairy, servings/day	1.8	1.6	1.7	1.7	1.6	0.1400
Total protein foods, servings/day	1.7	1.7	1.7	1.7	1.7	0.6149
Seafood and plant protein, servings/day	0.5	0.4	0.4	0.4	0.4	0.1272
Fatty acids, (PUFA+MUFA)/SFA	1.9	1.9	1.8	1.8	1.9	0.5742
Refined grains, servings/day	2.52	2.50	2.49	2.56	2.49	0.8896
Sodium, mg/day	2529	2461	2456	2472	2469	0.7621
Empty calories, % Energy	17.4	17.6	17.1	17.1	17.7	0.5536
Males, *n* = 2780						
Fruit and vegetables, servings/day	7.1	6.6	7.2	6.5	6.7	0.0780
Whole fruit, servings/day	2.0	1.7	2.0	1.9	1.9	0.1144
Greens and beans, servings/day	0.9	0.9	0.9	0.8	0.8	0.7477
Whole grains, servings/day	0.7	0.7	0.7	0.7	0.7	0.7435
Dairy, servings/day	1.7	1.6	1.6	1.7	1.7	0.7402
Total protein foods, servings/day	1.8	1.8	1.8	1.8	1.8	0.9663
Seafood and plant protein, servings/day	0.4	0.3	0.4	0.4	0.4	0.6525
Fatty acids, (PUFA + MUFA)/SFA	1.8	1.9	1.8	1.8	1.8	0.1392
Refined grains, servings/day	2.7	2.7	2.6	2.8	2.7	0.8035
Sodium, mg/day	2731	2686	2668	2667	2684	0.9741
Empty calories, % Energy	19.3	18.9	18.6	18.3	19.7	0.3036
Females, *n* = 3307						
Fruit and vegetables, servings/day	7.3	7.3	7.5	7.0	7.0	0.2536
Whole fruit, servings/day	2.5	2.3	2.6	2.3	2.3	0.0178
Greens and beans, servings/day	1.2	1.2	1.1	1.1	1.2	0.6539
Whole grains, servings/day	0.5	0.5	0.5	0.5	0.5	0.1575
Dairy, servings/day	1.8	1.6	1.7	1.7	1.6	0.1237
Total protein foods, servings/day	1.7	1.5	1.6	1.5	1.6	0.3253
Seafood and plant protein, servings/day	0.5	0.5	0.5	0.5	0.5	0.1339
Fatty acids, (PUFA+MUFA)/SFA	1.9	1.9	1.9	1.9	1.9	0.5697
Refined grains, servings/day	2.4	2.3	2.3	2.3	2.3	0.9232
Sodium, mg/day	2326	2242	2257	2263	2252	0.7842
Empty calories, % Energy	15.5	16.1	15.7	15.9	15.8	0.8226

PRS, polygenic risk score for obesity; HEI-C, Canadian adaptation of the Healthy Eating Index 2010.

**Table 5:** PRS and HEI-C main and interaction effects on adiposity-related outcomes, overall and by sex, among diet sample.

	Waist Circumference			BMI			% Body Fat		
	β	95% CI	*p*-Value	β	95% CI	*p*-Value	β	95% CI	*p*-Value
	Overall (*n* = 6087)								
PRS	1.3	(1.0, 1.6)	<0.0001	0.6	(0.5, 0.8)	<0.0001	0.6	(0.5, 0.8)	<0.0001
HEI-C	−1.6	(−2.0, −1.1)	<0.0001	−0.5	(−0.6, −0.3)	<0.0001	−0.7	(−0.9, −0.5)	<0.0001
PRS × HEI-C	−0.5	(−0.8, −0.1)	0.005	−0.1	(−0.3, 0.0)	0.0385	−0.2	(−0.3, 0.0)	0.0409
PRS in quintiles									
PRS Q 2	1.6	(0.6, 2.6)	0.0017	0.6	(0.2, 1.0)	0.0017	0.9	(0.3, 1.4)	0.0013
PRS Q 3	2.5	(1.5, 3.4)	<0.0001	1.1	(0.7, 1.5)	<0.0001	1.5	(0.9, 2.0)	<0.0001
PRS Q 4	2.8	(1.9, 3.8)	<0.0001	1.2	(0.8, 1.6)	<0.0001	1.5	(0.9, 2.0)	<0.0001
PRS Q 5	3.8	(2.8, 4.7)	<0.0001	1.8	(1.4, 2.2)	<0.0001	1.8	(1.2, 2.3)	<0.0001
HEI-C	−1.4	(−2.1, −0.6)	0.0004	−0.4	(−0.7, −0.1)	0.0047	−0.7	(0.3, −0.3)	0.0016
PRS Q 2 × HEI-C	0.6	(−0.4, 1.6)	0.2229	0.2	(−0.2, 0.6)	0.4019	0.3	(−0.1, −0.7)	0.3383
PRS Q 3 × HEI-C	−0.6	(−1.6, 0.5)	0.2875	−0.1	(−0.5, 0.3)	0.5676	−0.1	(0.3, −0.3)	0.6937
PRS Q 4 × HEI-C	−0.0	(−1.0, 1.0)	0.9623	0.1	(−0.3, 0.5)	0.6238	0.3	(−0.5, −1.0)	0.3130
PRS Q 5 × HEI-C	−0.9	(−1.9, 0.1)	0.0788	−0.3	(−0.7, 0.1)	0.1066	−0.5	(0.3, −0.3)	0.0663
Males (*n* = 2780)								
PRS	1.4	(1.0, 1.9)	<0.0001	0.6	(0.4, 0.8)	<0.0001	0.6	(0.3, 0.8)	<0.0001
HEI-C	−2.0	(−2.6, −1.4)	<0.0001	−0.6	(−0.8, −0.4)	<0.0001	−0.8	(−1.1, −0.5)	<0.0001
PRS × HEI-C	−0.8	(−1.2, −0.4)	0.0004	−0.3	(−0.4, −0.1)	0.0013	−0.3	(−0.6, −0.1)	0.0049
PRS in quintiles								
PRS Q 2	1.6	(0.2, 3.0)	0.0213	0.5	(0.0, 1.0)	0.0432	0.7	(−0.1, 1.4)	0.0669
PRS Q 3	3.0	(1.6, 4.4)	<0.0001	1.2	(0.7, 1.7)	<0.0001	1.4	(0.7, 2.1)	0.0002
PRS Q 4	2.7	(1.3, 4.1)	0.0001	1.2	(0.7, 1.7)	<0.0001	1.0	(0.3, 1.7)	0.0063
PRS Q 5	4.1	(2.7, 5.5)	<0.0001	1.7	(1.2, 2.2)	<0.0001	1.6	(0.9, 2.3)	<0.0001
HEI-C	−1.3	(−2.4, −0.3)	0.0136	−0.4	(−0.8, −0.0)	0.0465	−0.56	(−1.1, −0.0)	0.0403
PRS Q 2 × HEI-C	0.3	(−1.1, 1.7)	0.7006	0.1	(−0.4, 0.6)	0.6745	0.1	(−0.6, 0.9)	0.7365
PRS Q 3 × HEI-C	−0.8	(−2.2, 0.6)	0.2485	−0.2	(−0.7, 0.3)	0.4218	−0.3	(−1.0, 0.4)	0.4233
PRS Q 4 × HEI-C	−1.2	(−2.6, 0.2)	0.1001	−0.3	(−0.7, 0.33)	0.4858	−0.3	(−1.0, 0.4)	0.4112
PRS Q 5 × HEI-C	−1.6	(−3.0, −0.2)	0.0248	−0.6	(−1.1, −0.1)	0.0182	−0.7	(−1.5, −0.0)	0.0414
Females (*n* = 3307)								
PRS	1.2	(0.7, 1.6)	<0.0001	0.6	(0.5, 0.82)	<0.0001	0.7	(0.4, 0.9)	<0.0001
HEI-C	−1.2	(−1.8, −0.6)	<0.0001	−0.4	(−0.6, −0.15)	0.0013	−0.4	(−0.9, −0.3)	0.0005
PRS × HEI-C	−0.1	(−0.6, 0.3)	0.5953	−0.0	(−0.2, 0.15)	0.7248	−0.1	(−0.3, 0.2)	0.6448
PRS in quintiles								
PRS Q 2	1.4	(0.0, 2.8)	0.0496	0.7	(0.1, 1.29)	0.018	1.0	(0.2, 1.8)	0.0122
PRS Q 3	2.0	(0.6, 3.4)	0.005	1.1	(0.5, 1.69)	0.0002	1.5	(0.7, 2.3)	0.0002
PRS Q 4	2.8	(1.4, 4.2)	<0.0001	1.2	(0.6, 1.80)	<0.0001	1.8	(1.0, 2.6)	<0.0001
PRS Q 5	3.3	(1.9, 4.7)	<0.0001	1.8	(1.3, 2.42)	<0.0001	1.8	(1.0, 2.6)	<0.0001
HEI-C	−1.5	(−2.6, −0.5)	0.005	−0.5	(−0.9, −0.01)	0.0462	−0.7	(−1.3, −0.1)	0.0204
PRS Q 2 × HEI-C	0.9	(−0.5, 2.3)	0.1932	0.1	(−0.4, 0.70)	0.6507	0.3	(−0.5, 1.0)	0.5060
PRS Q 3 × HEI-C	0.1	(−1.4, 1.5)	0.9371	0.1	(−0.6, 0.66)	0.8653	0.2	(−0.7, 1.0)	0.7193
PRS Q 4 × HEI-C	0.9	(−0.6, 2.3)	0.2379	0.3	(−0.3, 0.89)	0.3233	0.7	(−0.1, 1.5)	0.0906
PRS Q 5 × HEI-C	−0.1	(−1.5, 1.3)	0.8514	−0.1	(−0.7, 0.45)	0.6602	−0.3	(−1.1, 0.5)	0.4351

Interaction term is depicted with an “×”. PRS Q1 (quintile 1) is the reference level for PRS in quintile analysis. β, estimate; PRS, polygenic risk score for obesity; Q2: quintile 2, Q3: quintile 3, Q4: quintile 4, Q5: quintile 5; Waist circumference in cm; BMI in kg/m^2^; HEI-C, Canadian adaptation of the Healthy Eating Index 2010; CI, confidence interval.

**Table 6:** Main and interaction effects of PRS and retail food environment scores on adiposity-related outcomes (n = 3718).

	Waist Circumference			BMI			% Body Fat			HEI-C		
	β	95% CI	*p*	β	95% CI	*p*	β	95% CI	*p*	β	95% CI	*p*
Display	−0.2	(−0.8, 0.3)	0.4192	−0.1	(−0.3, 0.1)	0.4526	−0.0	(−0.3, 0.2)	0.8276	0.0	(−0.3, 0.4)	0.8163
PRS	1.2	(0.8, 1.7)	<0.0001	0.6	(0.5, 0.8)	<0.0001	0.6	(0.4, 0.9)	<0.0001	0.0	(−0.5, 0.4)	0.9146
PRS × Display	0.3	(−0.2, 0.8)	0.1752	0.1	(−0.1, 0.2)	0.5969	0.2	(−0.1, 0.4)	0.2485	0.2	(−0.2, 0.6)	0.2679
Discount	0.3	(−0.2, 0.8)	0.2403	0.1	(−0.1, 0.3)	0.2588	0.1	(−0.1, 0.4)	0.3292	0.0	(−0.3, 0.4)	0.8221
PRS	1.2	(0.8, 1.7)	<0.0001	0.7	(0.5, 0.8)	<0.0001	0.6	(0.4, 0.9)	<0.0001	0.0	(−0.4, 0.3)	0.8077
PRS × Discount	−0.4	(−1.0, 0.1)	0.117	−0.1	(−0.3, 0.1)	0.2439	−0.2	(−0.4, 0.1)	0.2853	0.2	(−0.3, 0.4)	0.6758
Regular price	0.1	(−0.5, 0.8)	0.6463	0.1	(−0.1, 0.4)	0.3351	0.1	(−0.2, 0.4)	0.4904	0.0	(−0.3, 0.4)	0.7919
PRS	1.2	(0.8, 1.7)	<0.0001	0.6	(0.5, 0.8)	<0.0001	0.6	(0.4, 0.9)	<0.0001	−0.3	(−0.7, 0.1)	0.1128
PRS × Regular price	0.7	(0.1, 1.3)	0.0308	0.1	(−0.1, 0.4)	0.10661	0.2	(−0.1,0.5)	0.1838	0.2	(−0.2, 0.6)	0.2456
Variety	−0.1	(−0.5, 0.4)	0.7351	−0.1	(−0.3, 0.1)	0.2864	−0.1	(−0.3, 0.2)	0.526	0.0	(−0.3, 0.4)	0.7959
PRS	1.2	(0.8, 1.7)	<0.0001	0.7	(0.5, 0.8)	<0.0001	0.6	(0.4, 0.9)	<0.0001	−0.2	(−0.5, 0.2)	0.3372
PRS × Variety	−0.2	(−0.7, 0.2)	0.2598	−0.1	(−0.2, 0.1)	0.3436	−0.1	(−0.3, 0.1)	0.2676	−0.1	(−0.5, 0.3)	0.5553

Interaction term is depicted with an “×”. β, estimate; CI, confidence interval; *p*, *p*-value; PRS, polygenic risk score for obesity; Waist circumference in cm; BMI in kg/m^2^; HEI-C, Canadian adaptation of the Healthy Eating Index 2010. The retail measurement scores indicate a ratio of vegetables to soft drinks. Display, percentage of Stock Keeping Units (SKUs) on display at a given time; Discount, frequency of price promotion; Regular price, regular price per serving; Variety, number of distinct SKUs at point of purchase.

**Table 7:** Main and interaction effects of PRS and retail food environment scores on adiposity-related outcomes among males (*n* = 1726).

	Waist Circumference			BMI			% of Body Fat			HEI-C		
	β	95% CI	*p*	β	95% CI	*p*	β	95% CI	*p*	β	95% CI	*p*
Display	−0.8	(−1.5, −0.1)	0.0222	−0.3	(−0.5, −0.0)	0.033	−0.3	(−0.7, 0.0)	0.0787	−0.3	(−0.9, 0.3)	0.3511
PRS	1.3	(0.7, 1.8)	<0.0001	0.6	(0.4, 0.8)	<0.0001	0.6	(0.3, 0.9)	<0.0001	−0.1	(−0.6, 0.5)	0.8711
PRS × Display	0.5	(−0.3, 1.3)	0.2177	0.1	(−0.1, 0.4)	0.2995	0.2	(−0.2, 0.5)	0.4214	0.0	(−0.5, 0.6)	0.9593
Discount	0.2	(−0.5, 0.8)	0.6425	0.0	(−0.2, 0.2)	0.9398	0.0	(−0.3, 0.3)	0.8701	−0.3	(−0.8, 0.2)	0.2508
PRS	1.3	(0.8, 1.9)	<0.0001	0.6	(0.5, 0.8)	<0.0001	0.6	(0.4, 0.9)	<0.0001	−0.1	(−0.6, 0.5)	0.8043
PRS × Discount	−1.2	(−1.9, −0.5)	0.0005	−0.4	(−0.7, −0.2)	0.0012	−0.5	(−0.9, −0.1)	0.0131	0.1	(−0.5, 0.8)	0.6485
Regular price	0.4	(−0.5, 1.3)	0.3922	0.1	(−0.2, 0.4)	0.3681	0.2	(−0.2, 0.6)	0.2868	0.0	(−0.6, 0.6)	0.9962
PRS	1.3	(0.7, 1.8)	<0.0001	0.6	(0.4, 0.8)	<0.0001	0.6	(0.3, 0.9)	<0.0001	−0.1	(−0.6, 0.5)	0.8386
PRS × Regular price	0.6	(−0.1, 1.4)	0.108	0.1	(−0.2, 0.4)	0.522	0.0	(−0.4, 0.4)	0.9947	0.2	(−0.3, 0.7)	0.4235
Variety	0.4	(−0.2, 1.2)	0.1622	0.1	(−0.1, 0.3)	0.4879	0.1	(−0.3, 0.4)	0.6632	−0.1	(−0.6, 0.4)	0.6045
PRS	1.3	(0.7, 1.8)	<0.0001	0.6	(0.4, 0.8)	<0.0001	0.6	(0.3, 0.9)	<0.0001	−0.0	(−0.6, 0.5)	0.8737
PRS × Variety	−0.1	(−0.6, 0.5)	0.8526	−0.0	(−0.2, 0.2)	0.7518	−0.1	(−0.4, 0.2)	0.5731	−0.3	(−0.8, 0.3)	0.3926

Interaction term is depicted with an “×”. β, estimate; CI, confidence interval; *p*, *p*-value; PRS, polygenic risk score for obesity; Waist circumference in cm; BMI in kg/m^2^; HEI-C, Canadian adaptation of the Healthy Eating Index 2010. The retail measurement scores indicate a ratio of vegetables to soft drinks. Display, percentage of Stock Keeping Units (SKUs) on display at a given time; Discount, frequency of price promotion; Regular price, regular price per serving; Variety, number of distinct SKUs at point of purchase.

**Table 8:** Main and interaction effects of genetic risk score and retail food environment scores on adiposity-related outcomes among females (*n* = 1992).

	Waist Circumference			BMI			% Body Fat			HEI-C		
	β	95% CI	*p*	β	95% CI	*p*	β	95% CI	*p*	β	95% CI	*p*
Display	0.3	(−0.5, 1.0)	0.5092	0.1	(−0.2, 0.4)	0.49	0.2	(−0.2, 0.6)	0.2978	0.2	(−0.4, 0.7)	0.5372
PRS	1.1	(0.5, 1.7)	0.0002	0.7	(0.4, 0.9)	<0.0001	0.7	(0.3, 1.0)	<0.0001	0.1	(−0.3, 0.5)	0.6653
PRS × Display	0.4	(−0.4, 1.2)	0.3181	0.0	(−0.3, 0.4)	0.7923	0.2	(−0.2, 0.6)	0.2412	0.2	(−0.3, 0.7)	0.4584
Discount	0.5	(−0.2, 1.1)	0.1735	0.2	(−0.2, 0.4)	0.1351	0.0	(−0.3, 0.3)	0.8701	0.1	(−0.4, 0.6)	0.6542
PRS	1.1	(0.5, 1.8)	0.0002	0.7	(0.4, 0.9)	<0.0001	0.6	(0.4, 0.9)	<0.0001	0.1	(−0.3, 0.5)	0.6439
PRS × Discount	0.2	(−0.7, 1.1)	0.6592	0.1	(−0.3, 0.5)	0.6451	0.1	(−0.4, 0.5)	0.705	0.1	(−0.5, 0.7)	0.7453
Regular price	0.0	(−0.7, 0.8)	0.9732	0.1	(−0.2, 0.4)	0.4321	0.2	(−0.1, 0.5)	0.1952	−0.6	(−1.1, −0.0)	0.0406
PRS	1.1	(0.5, 1.7)	0.0002	0.7	(0.4, 0.9)	<0.0001	0.7	(0.4, 1.0)	<0.0001	0.1	(−0.3, 0.5)	0.6051
PRS × Regular price	0.6	(−0.2, 1.3)	0.1185	0.2	(−0.1, 0.4)	0.3005	0.4	(0.0, 0.8)	0.0489	0.0	(−0.5, 0.6)	0.8814
Variety	−0.5	(−1.0, 0.1)	0.0807	−0.2	(−0.5, 0.0)	0.0523	−0.2	(−0.5, 0.1)	0.1727	−0.1	(−0.6, 0.3)	0.5254
PRS	1.2	(0.5, 1.8)	0.0002	0.7	(0.4, 0.9)	<0.0001	0.7	(0.4, 1.0)	<0.0001	0.1	(−0.3, 0.5)	0.6498
PRS × Variety	−0.4	(−0.9, 0.2)	0.2002	−0.1	(−0.4, 0.1)	0.3209	−0.2	(−0.5, 0.1)	0.3057	0.0	(−0.4, 0.5)	0.9252

Interaction term is depicted with an “×”. β, estimate; CI, confidence interval; *p*, *p*-value; PRS, polygenic risk score for obesity; Waist circumference in cm; BMI in kg/m^2^; HEI-C, Canadian adaptation of the Healthy Eating Index 2010. The retail measurement scores indicate a ratio of vegetables to soft drinks. Display, percentage of Stock Keeping Units (SKUs) on display at a given time; Discount, frequency of price promotion; Regular price, regular price per serving; Variety, number of distinct SKUs at point of purchase.

## Supplementary Materials

The following are available online at https://www.mdpi.com/2072-6643/12/11/3349/s1, Figure S1: Participant flow chart, Figure S2: CARTaGENE FSAs represented in Quebec retail food environment data, Table S1: genetic variants associated with body mass index, Table S2: sensitivity analysis for genetic risk score and diet quality score main and interaction effects on waist circumference, body mass index, and body fat percent, Table S3: participant characteristics of retail analysis sample, Table S4: mean and standard deviation of HEI-C score, waist circumference and BMI of PRS quintile in retail analysis sample, Table S5: mean of HEI-C individual components by PRS quintile in retail analysis sample, Table S6: 23 genes variants identified by Locke et al. are among the top 25 BMI susceptibility genes with highest expression in brain regions (insula and substantia nigra).
