# Supplementary material for: Diet Quality and Food Prices Modify Associations between Genetic Susceptibility to Obesity and Adiposity Outcomes
Source: Nutrients. 2020 Oct 30;12(11):3349. doi: 10.3390/nu12113349 (PMC7692602; doi:10.3390/nu12113349)
Supplement: Supplementary file 1 [file nutrients-12-03349-s001.pdf]

Supplementary Material

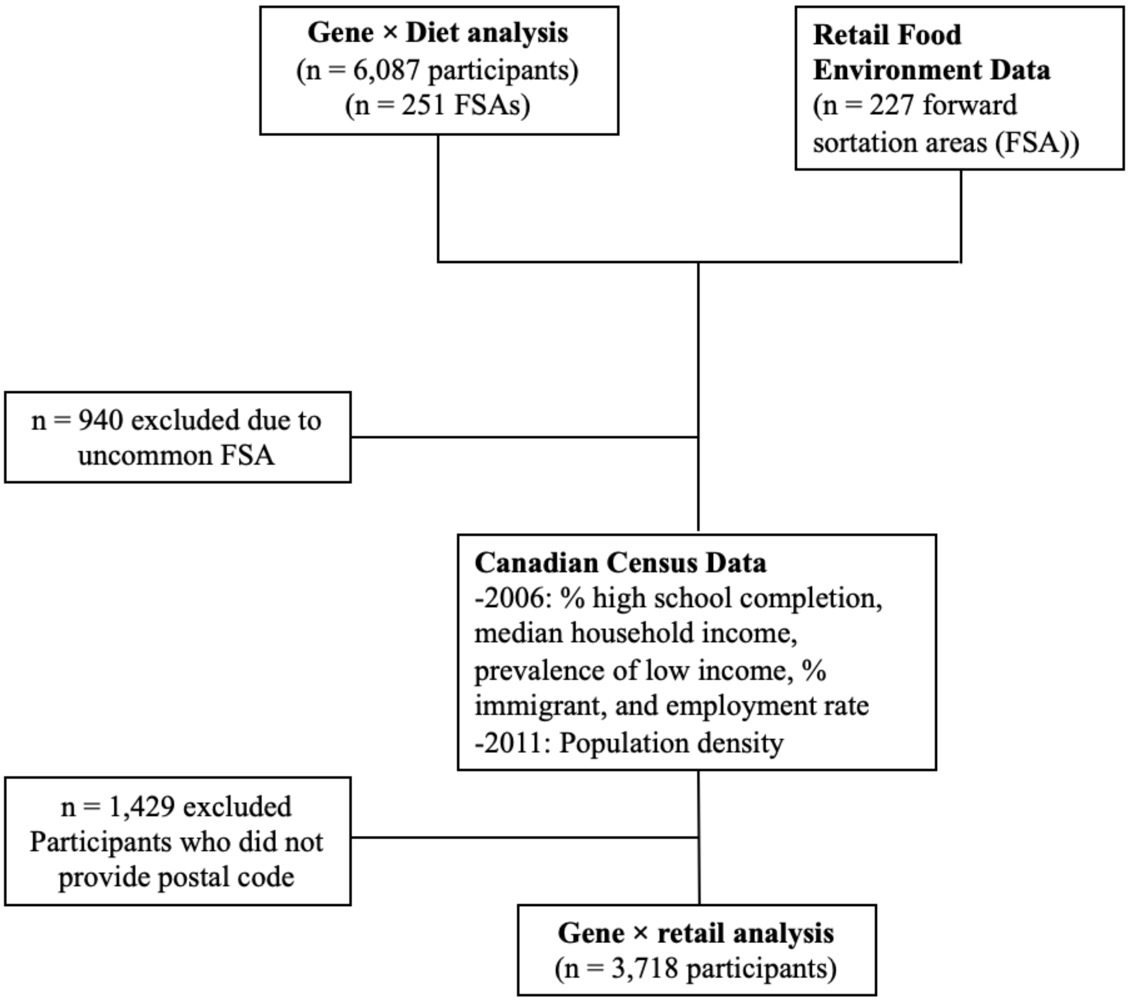

Figure S1: Participant flow chart

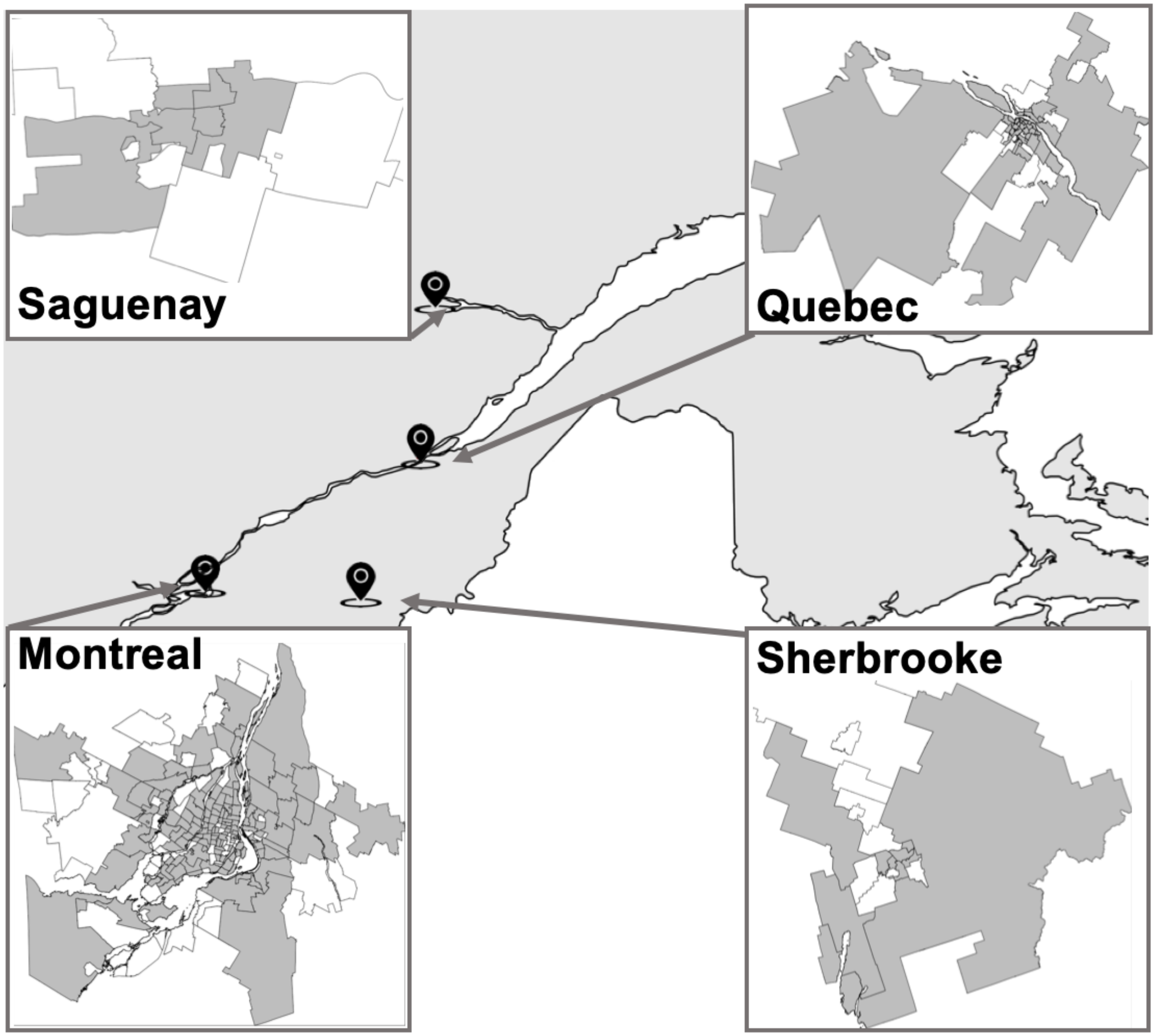

Figure S2: CARTaGENE FSAs (shaded in grey for each regions) represented in Quebec retail food environment data.

Supplemental Table S1: Genetic variants associated with body mass index (1)

SNP ID	SNP	Chr	Position	Nearest genes	BMI-increasing allele	Effect size
77 loci reaching genome-wide significance in European-descent individuals						
1	rs657452	1	49,362,434	*AGBL4*	A	0.0227
2	rs11583200	1	50,332,407	*ELAVL4*	C	0.0177
3	rs12566985	1	74,774,781	*FPGT*	G	0.0242
4	rs12401738	1	78,219,349	*FUBP1*	A	0.0211
5	rs17024393	1	109,956,211	*GNAT2*	C	0.0658
6	rs2820292	1	200,050,910	*NAV1*	C	0.0195
7	rs3101336	1	72,523,773	*NEGR1*	C	0.0334
8	rs11165643	1	96,696,685	*PTBP2*	T	0.0218
9	rs543874	1	176,156,103	*SEC16B*	G	0.0482
10	rs10182181	2	25,003,800	*ADCY3*	G	0.0307
11	rs11688816	2	62,906,552	*EHBP1*	G	0.0172
12	rs7599312	2	213,121,476	*ERBB4*	G	0.0220
13	rs11126666	2	26,782,315	*KCNK3*	A	0.0207
14	rs2121279	2	142,759,755	*LRP1B*	T	0.0245
15	rs13021737	2	622,348	*TMEM18*	G	0.0601
16	rs1528435	2	181,259,207	*UBE2E3*	T	0.0178
17	rs13078960	3	85,890,280	*CADM2*	G	0.0297
18	rs1516725	3	187,306,698	*ETV5*	C	0.0451
19	rs2365389	3	61,211,502	*FHIT*	C	0.0200
20	rs3849570	3	81,874,802	*GBE1*	A	0.0188
21	rs6804842	3	25,081,441	*RARB*	G	0.0185
22	rs16851483	3	142,758,126	*RASA2*	T	0.0483
23	rs10938397	4	44,877,284	*GNPDA2*	G	0.0402
24	rs11727676	4	145,878,514	*HHIP*	T	0.0358
25	rs17001654	4	77,348,592	*SCARB2*	G	0.0306
26	rs13107325	4	103,407,732	*SLC39A8*	T	0.0477
27	rs2112347	5	75,050,998	*POC5*	T	0.0261
28	rs205262	6	34,671,142	*C6orf106*	G	0.0221
29	rs9400239	6	109,084,356	*FOXO3*	C	0.0188
30	rs13191362	6	162,953,340	*PARK2*	A	0.0277
31	rs2033529	6	40,456,631	*TDRG1*	G	0.0190
32	rs2207139	6	50,953,449	*TFAP2B*	G	0.0447
33	rs1167827	7	75,001,105	*HIP1*	G	0.0202
34	rs17405819	8	76,969,139	*HNF4G*	T	0.0224
35	rs2033732	8	85,242,264	*RALYL*	C	0.0192

36	rs4740619	9	15,624,326	*C9orf93*	T	0.0179
37	rs6477694	9	110,972,163	*EPB41L4B*	C	0.0174
38	rs10968576	9	28,404,339	*LINGO2*	G	0.0249
39	rs10733682	9	128,500,735	*LMX1B*	A	0.0174
40	rs1928295	9	119,418,304	*TLR4*	T	0.0188
41	rs7899106	10	87,400,884	*GRID1*	G	0.0395
42	rs17094222	10	102,385,430	*HIF1AN*	C	0.0249
43	rs11191560	10	104,859,028	*NT5C2*	C	0.0308
44	rs7903146	10	114,748,339	*TCF7L2*	C	0.0234
45	rs11030104	11	27,641,093	*BDNF*	A	0.0414
46	rs12286929	11	114,527,614	*CADM1*	G	0.0217
47	rs2176598	11	43,820,854	*HSD17B12*	T	0.0198
48	rs3817334	11	47,607,569	*MTCH2*	T	0.0262
49	rs4256980	11	8,630,515	*TRIM66*	G	0.0209
50	rs7138803	12	48,533,735	*BCDIN3D*	A	0.0315
51	rs11057405	12	121,347,850	*CLIP1*	G	0.0307
52	rs12016871	13	26,915,782	*MTIF3*	T	0.0298
53	rs12429545	13	53,000,207	*OLFM4*	A	0.0334
54	rs7141420	14	78,969,207	*NRXN3*	T	0.0235
55	rs11847697	14	29,584,863	*PRKD1*	T	0.0492
56	rs12885454	14	28,806,589	*PRKD1*	C	0.0207
57	rs10132280	14	24,998,019	*STXBP6*	C	0.0230
58	rs3736485	15	49,535,902	*DMXL2*	A	0.0176
59	rs16951275	15	65,864,222	*MAP2K5*	T	0.0311
60	rs3888190	16	28,796,987	*ATP2A1*	A	0.0309
61	rs1558902	16	52,361,075	*FTO*	A	0.0818
62	rs12446632	16	19,842,890	*GPRC5B*	G	0.0403
63	rs9925964	16	31,037,396	*KAT8*	A	0.0192
64	rs758747	16	3,567,359	*NLRC3*	T	0.0225
65	rs2650492	16	28,240,912	*SBK1*	A	0.0207
66	rs1000940	17	5,223,976	*RABEP1*	G	0.0192
67	rs12940622	17	76,230,166	*RPTOR*	G	0.0182
68	rs1808579	18	19,358,886	*C18orf8*	C	0.0167
69	rs7243357	18	55,034,299	*GRP*	T	0.0217
70	rs6567160	18	55,980,115	*MC4R*	C	0.0556
71	rs29941	19	39,001,372	*KCTD15*	G	0.0182
72	rs17724992	19	18,315,825	*PGPEP1*	A	0.0194
73	rs2287019	19	50,894,012	*QPCTL*	C	0.0360
74	rs2075650	19	50,087,459	*TOMM40*	A	0.0258
75	rs3810291	19	52,260,843	*ZC3H4*	A	0.0283

76	rs1016287	2	59,159,129	*LINC01122*	T	0.0229
77	rs2245368	7	76,446,079	*DTX2P1*	C	0.0317
20 loci reaching genome-wide significance with inclusion of non-European-decent individuals						
78	rs977747	1	47,457,264	*TAL1*	T	0.0167
79	rs17203016	2	207,963,763	*CREB1*	G	0.0210
80	rs1460676	2	164,275,935	*FIGN*	C	0.0197
81	rs2176040	2	226,801,046	*LOC646736*	A	0.0141
82	rs492400	2	219,057,996	*USP37*	C	0.0158
83	rs7715256	5	153,518,086	*GALNT10*	G	0.0163
84	rs13201877	6	137,717,234	*IFNGR1*	G	0.0233
85	rs9374842	6	120,227,364	*LOC285762*	T	0.0187
86	rs6465468	7	95,007,450	*ASB4*	T	0.0166
87	rs9641123	7	93,035,668	*CALCR*	C	0.0191
88	rs16907751	8	81,538,012	*ZBTB10*	C	0.0350
89	rs1441264	13	78,478,920	*MIR548A2*	A	0.0175
90	rs9540493	13	65,103,705	*MIR548X2*	A	0.0172
91	rs7164727	15	70,881,044	*LOC100287559*	T	0.0180
92	rs2080454	16	47,620,091	*CBLN1*	C	0.0168
93	rs4787491	16	29,922,838	*INO80E*	G	0.0159
94	rs9914578	17	1,951,886	*SMG6*	G	0.0201
95	rs7239883	18	38,401,669	*LOC284260*	G	0.0164
96	rs6091540	20	50,521,269	*ZFP64*	C	0.0188
97	rs2836754	21	39,213,610	*ETS2*	C	0.0164

SNP, single nuclear polymorphism; Chr, chromosome

Table S2: Sensitivity analysis (excluding total energy intake from the model) for genetic risk score and diet quality score main and interaction effects on waist circumference, body mass index, and body fat percent

	**Waist circumference (cm)**			**Body Mass Index (kg/m2)**			**Percentage of body fat**		
	Estimate	95% CI	p-value	Estimate	95% CI	p-value	Estimate	95% CI	p-value
				Overall (*n* = 6,087)					
PRS	1.4	(1.0, 1.7)	<.0001	0.6	(0.5, 0.8)	<.0001	0.6	(0.5, 0.8)	<.0001
HEI-C	0.2	(-0.2, 0.6)	0.33	0.2	(0.0, 0.3)	0.06	0.1	(-0.1, 0.3)	0.31
PRS×HEI-C	-0.4	(-0.8, -0.1)	0.01	-0.1	(-0.3, 0.0)	0.05	-0.2	(-0.3, 0.0)	0.05
PRS in quintiles									
PRS quintile 2	1.8	(0.8, 2.8)	0.01	0.7	(0.3, 1.1)	0.01	1.0	(0.4, 1.5)	0.01
PRS quintile 3	2.6	(1.4, 3.6)	<.0001	1.2	(0.8, 1.6)	<.0001	1.5	(1.0, 2.1)	<.0001
PRS quintile 4	3.1	(2.0, 4.1)	<.0001	1.3	(0.9, 1.7)	<.0001	1.6	(1.0, 2.1)	<.0001
PRS quintile 5	3.9	(2.9, 4.9)	<.0001	1.8	(1.4, 2.3)	<.0001	1.8	(1.3, 2.4)	<.0001
HEI-C	0.4	(-0.4, 1.1)	0.33	0.1	(-0.3, 0.5)	0.21	0.1	(-0.3, 0.5)	0.51
PRS quintile 2×HEI-C	0.5	(-0.5, 1.5)	0.32	0.1	(-0.3, 0.5)	0.51	0.2	(-0.3, 0.8)	0.43
PRS quintile 3×HEI-C	-0.5	(-1.6, 0.5)	0.34	-0.1	(-0.5, 0.3)	0.62	-0.1	(-0.7, 0.5)	0.74
PRS quintile 4×HEI-C	-0.0	(-1.1, 1.0)	0.98	0.1	(-0.3, 0.5)	0.62	0.3	(-0.3, 0.8)	0.31
PRS quintile 5×HEI-C	-0.9	(-1.2, 0.1)	0.08	-0.3	(-0.7, 0.1)	0.11	-0.5	(-1.0, 0.0)	0.07
				Males (*n* = 2,780)					
PRS	1.5	(1.1, 2.0)	<.0001	0.6	(0.5, 0.8)	<.0001	0.6	(0.4, 0.8)	<.0001
HEI-C	0.2	(-0.3, 0.8)	0.41	0.2	(0.0, 0.3)	0.11	0.1	(-0.2, 0.4)	0.57
PRS×HEI-C	-0.7	(-1.2, -0.3)	0.01	-0.2	(-0.4, -0.1)	0.01	-0.3	(-0.5, -0.1)	0.01
PRS in quintiles									
PRS quintile 2	1.9	(0.5, 3.4)	0.01	0.6	(0.1, 1.1)	0.02	0.8	(0.1, 1.5)	0.03
PRS quintile 3	3.3	(1.8, 4.7)	<.0001	1.2	(0.7, 1.8)	<.0001	1.5	(0.7, 2.2)	<.0001
PRS quintile 4	3.1	(1.6, 4.5)	<.0001	1.3	(0.8, 1.8)	<.0001	1.2	(0.4, 1.9)	<0.01
PRS quintile 5	4.5	(3.0, 5.9)	<.0001	1.8	(1.3, 2.3)	<.0001	1.8	(1.0, 2.5)	<.0001
HEI-C	0.9	(-0.2, 1.9)	0.11	0.3	(-0.1, 0.7)	0.10	0.3	(-0.2, 0.9)	0.25
PRS quintile 2×HEI-C	0.2	(-1.3, 1.7)	0.80	0.1	(-0.4, 0.6)	0.76	0.1	(-0.7, 0.8)	0.81
PRS quintile 3×HEI-C	-0.9	(-2.2, 0.7)	0.29	-0.2	(-0.7, 0.3)	0.46	-0.3	(-1.0, 0.5)	0.46
PRS quintile 4×HEI-C	-1.2	(-2.7, 0.3)	0.11	-0.2	(-0.7, 0.3)	0.49	-0.3	(-1.1, 0.4)	0.41
PRS quintile 5×HEI-C	-1.4	(-2.9, 0.0)	0.05	-0.6	(-1.1, -0.0)	0.04	-0.7	(-1.4, 0.1)	0.07
				Females (*n* = 3,307)					

PRS	1.2	(0.7, 1.6)	<.0001	0.6	(0.5, 0.8)	<.0001	0.7	(0.4, 0.9)	<.0001
HEI-C	0.2	(-0.3, 0.8)	0.40	0.2	(-0.1, 0.4)	0.14	0.2	(-0.1, 0.5)	0.29
PRS×HEI-C	-0.2	(-0.6, 0.3)	0.52	-0.0	(-0.2, 0.1)	0.65	-0.1	(-0.3, 0.2)	0.57
PRS in quintiles									
PRS quintile 2	1.5	(0.0, 2.9)	0.044	0.7	(0.1, 1.3)	0.016	1.0	(0.2, 1.8)	0.011
PRS quintile 3	2.2	(0.7, 3.6)	<.001	1.2	(0.6, 1.8)	<.001	1.6	(0.8, 2.4)	<.001
PRS quintile 4	2.9	(1.5, 4.4)	<.0001	1.3	(0.7, 1.9)	<.0001	1.9	(1.1, 2.6)	<.0001
PRS quintile 5	3.3	(1.9, 4.7)	<.0001	1.9	(1.3, 2.4)	<.0001	1.8	(1.0, 2.6)	<.0001
HEI-C	-0.1	(-1.2, 1.0)	0.84	0.1	(-0.3, 0.5)	0.65	0.0	(-0.6, 0.6)	0.95
PRS quintile 2×HEI-C	1.0	(-0.4, 2.4)	0.18	0.2	(-0.4, 0.7)	0.61	0.3	(-0.5, 1.1)	0.47
PRS quintile 3×HEI-C	0.1	(-1.4, 1.6)	0.95	0.1	(-0.6, 0.7)	0.88	0.1	(-0.7, 1.0)	0.73
PRS quintile 4×HEI-C	0.9	(-0.6, 2.4)	0.22	0.3	(-0.3, 0.9)	0.30	0.7	(-0.1, 1.5)	0.08
PRS quintile 5×HEI-C	-0.2	(-1.6, 1.2)	0.77	-0.2	(-0.8, 0.4)	0.60	-0.3	(-1.1, 0.4)	0.39

Table S3: Participant characteristics of retail analysis sample (*n* = 3,718)

**Characteristic**	**Descriptive statistics**[a]
Age, years	55(8)
Female, n (%)	1992 (53.58)
Household Income, (n (%))	
<CAD 25,000	288 (7.75)
CAD 25,000-50,000	814 (21.89)
CAD 50,000-75,000	823 (22.14)
CAD 75,000-150,000	1258 (33.84)
>CAD 150,000	406 (10.92)
Missing	129 (3.47)
Education, (n (%))	
High school or less	789 (21.22)
College	1195 (32.14)
University or higher	1726 (46.42)
Missing	8 (0.22)
Language (French), n (%)	3569 (95.99)
Ethnicity (Caucasian), n(%)	3536 (96.30)
Physical activity level	1.48 (0.36)
Smoking status, n (%)	
Never	1522 (40.94)
Daily	422 (11.35)
Occasionally	127 (3.42)
Past	1641 (44.14)
Missing	6 (0.16)
Marital status, n (%)	
Married	2480 (66.70)
Divorced	712 (19.15)
Single	520 (13.99)
Missing	6 (0.16)
Season, n (%)	
October - March	2183 (58.71)
April - September	1535 (41.29)
Waist Circumference (cm)	93.7 (14.6)
BMI	27.6 (5.4)
Percentage of body fat	30.9 (8.7)
Genetic risk score	87.31 (6.34)
Energy intake (kcal/day)	1717(941)
Alcohol consumption (kcal/day)	83 (182)
Reporter status, n (%)	
Under reporter	1705 (45.86)
Plausible reporter	1808 (48.63)
Over reporter	205 (5.51)
Diet quality scores (min.-max.)	
HEI-C 2010 score (0-100)	57.6 (13.2)
Adequacy sub-score (0-60)	32.4(12.0)

Moderate sub-score (0-40)	25.2 (5.2)
Retail measurement (min.-max.)	
Display measure (0-42.50)	1.16 (4.47)
Discount measure (0-6.53)	1.96 (1.41)
Regular price measure (0.12-10.17)	1.55 (2.05)
Variety measure (0-3.37)	0.93 (0.99)

[a]Values are mean (standard deviation) unless otherwise indicated

Table S4: Mean and standard deviation of HEI-C score, waist circumference and BMI of PRS quintile in retail analysis sample

**PRS quintile**	**1**	**2**	**3**	**4**	**5**	**p-value for trend**
Combined, *n* = 3,718						
HEI-C	58.1	57.0	57.3	57.4	57.2	0.52
Waist circumference (cm)	91.8	93.5	94.4	94.6	95.8	<.0001
BMI (kg/m^2)	26.6	27.3	27.7	28.0	28.6	<.0001
Percentage of body fat	29.3	30.2	30.7	30.8	31.2	<.0001
Male, *n* = 1,726						
HEI-C	55.0	54.6	54.5	56.1	53.9	0.29
Waist circumference (cm)	97.1	99.2	99.9	99.8	101.3	<.001
BMI (kg/m^2)	27.1	27.7	28.3	28.5	28.8	<.0001
Percentage of body fat	24.3	25.2	25.7	25.5	26.1	0.006
Female, *n* = 1,992						
HEI-C	61.1	59.5	60.0	59.0	60.4	0.14
Waist circumference (cm)	86.5	87.8	88.8	89.7	90.3	<.001
BMI (kg/m^2)	26.1	26.9	27.2	27.5	28.2	<.0001
Percentage of body fat	34.2	35.2	35.8	36.2	36.2	<.001

Mean values are adjusted for sex (combined sample only) and age.

HEI-C, Canadian adaptation of the Healthy Eating Index 2010; BMI, Body Mass Index; PRS, polygenic risk score for obesity

Table S5: Mean of HEI-C individual components by PRS quintile in retail analysis sample

PRS quintile	1	2	3	4	5	p-value for trend
Combined, *n* = 3,718						
Fruit and vegetables, servings/day	7.2	7.0	7.1	6.9	6.7	0.32
Whole fruit, servings/day	2.3	2.0	2.2	2.2	2.0	0.01
Greens and beans, servings/day	1.0	1.1	1.0	1.0	1.0	0.30
Whole grains, servings/day	0.6	0.6	0.6	0.6	0.6	0.62
Dairy, servings/day	1.8	1.6	1.6	1.7	1.6	0.23
Total protein foods, servings/day	1.8	1.7	1.7	1.7	1.7	0.77
Seafood and plant protein, servings/day	0.5	0.4	0.4	0.4	0.4	0.14
Fatty acids, (PUFA+MUFA)/SFA	1.9	1.9	1.8	1.8	1.8	0.77
Refined grains, servings/day	2.5	2.6	2.5	2.6	2.4	0.71
Sodium, mg/day	2533	2517	2446	2448	2441	0.63
Empty calories, % Energy	17.1	18.1	17.7	16.8	17.7	0.14
Male, *n* = 1,726						
Fruit and vegetables, servings/day	6.9	6.7	6.9	6.6	6.4	0.48
Whole fruit, servings/day	2.0	1.7	2.0	2.0	1.8	0.11
Greens and beans, servings/day	0.8	0.9	0.9	0.9	0.9	0.50
Whole grains, servings/day	0.7	0.7	0.8	0.7	0.7	0.91
Dairy, servings/day	1.6	1.6	1.6	1.6	1.6	0.95
Total protein foods, servings/day	1.7	1.8	1.8	1.9	1.8	0.87
Seafood and plant protein, servings/day	0.4	0.3	0.4	0.4	0.4	0.73
Fatty acids, (PUFA+MUFA)/SFA	1.8	1.9	1.8	1.8	1.8	0.46
Refined grains, servings/day	2.7	2.7	2.6	2.8	2.6	0.80
Sodium, mg/day	2652	2696	2642	2671	2602	0.96
Empty calories, % Energy	19.6	19.6	18.9	17.7	20.0	0.10
Female, *n* = 1,992						
Fruit and vegetables, servings/day	7.5	7.3	7.3	7.1	7.0	0.78
Whole fruit, servings/day	2.6	2.3	2.5	2.3	2.3	0.09
Greens and beans, servings/day	1.3	1.2	1.1	1.1	1.2	0.12
Whole grains, servings/day	0.6	0.5	0.5	0.4	0.5	0.04
Dairy, servings/day	1.9	1.6	1.7	1.7	1.7	0.21
Total protein foods, servings/day	1.8	1.6	1.6	1.5	1.6	0.08
Seafood and plant protein, servings/day	0.6	0.5	0.5	0.5	0.5	0.07
Fatty acids, (PUFA+MUFA)/SFA	1.9	1.9	1.9	1.9	1.9	0.94
Refined grains, servings/day	2.4	2.5	2.3	2.3	2.3	0.63
Sodium, mg/day	2407	2343	2249	2234	2278	0.35
Empty calories, % Energy	14.8	16.5	16.5	15.9	15.5	0.08

Mean values are adjusted for sex (combined sample only) and age.
HEI-C, Canadian adaptation of the Healthy Eating Index 2010; PRS, polygenic risk score for obesity

Table S6: 23 genes variants identified by Locke et al.[1] are among the top 25 BMI susceptibility genes with highest expression in brain regions (insula and substantia nigra)[2].

SNP ID	SNP	Chr	Nearest genes	Effect-allele on BMI
1	rs657452	1	*AGBL4*	G
2	rs7138803	12	*BCDIN3D*	A
3	rs205262	6	*C6orf106*	G
4	rs13078960	3	*CADM2*	G
5	rs11583200	1	*ELAVL4*	T
6	rs7599312	2	*ERBB4*	G
7	rs1516725	3	*ETV5*	C
8	rs9400239	6	*FOXO3*	C
9	rs1558902	16	*FTO*	A
10	rs7899106	10	*GRID1*	G
11	rs11727676	4	*HHIP*	T
12	rs1167827	7	*HIP1*	G
13	rs2121279	2	*LRP1B*	T
14	rs16951275	15	*MAP2K5*	T
15	rs2820292	1	*NAV1*	C
16	rs3101336	1	*NEGR1*	C
17	rs7141420	14	*NRXN3*	T
18	rs11191560	10	*NT5C2*	C
19	rs1000940	17	*RABEP1*	G
20	rs2033732	8	*RALYL*	C
21	rs10132280	14	*STXBP6*	C
22	rs2075650	19	*TOMM40*	A
23	rs1528435	2	*UBE2E3*	T

SNP, single nuclear polymorphism; Chr, chromosome

1. Locke, A.E.; Kahali, B.; Berndt, S.I.; Justice, A.E.; Pers, T.H.; Day, F.R.; Powell, C.; Vedantam, S.; Buchkovich, M.L.; Yang, J., et al. Genetic studies of body mass index yield new insights for obesity biology. *Nature* **2015**, *518*, 197-206, doi:10.1038/nature14177.
2. Ndiaye, F.K.; Huyvaert, M.; Ortalli, A.; Canouil, M.; Lecoeur, C.; Verbanck, M.; Lobbens, S.; Khamis, A.; Marselli, L.; Marchetti, P., et al. The expression of genes in top obesity-associated loci is enriched in insula and substantia nigra brain regions involved in addiction and reward. *Int J Obes (Lond)* **2020**, *44*, 539-543, doi:10.1038/s41366-019-0428-7.
